# Thoracoscopic esophageal drainage for tracheal compression due to mucocele after esophagogastric bypass: a case report

**DOI:** 10.1186/s40792-023-01693-w

**Published:** 2023-06-15

**Authors:** Takeshi Yamashita, Koji Otsuka, Satoru Goto, Tomotake Ariyoshi, Kentaro Motegi, Masahiro Kohmoto, Akira Saito, Koichiro Fujimasa, Yoshihito Sato, Rei Kato, Tetsuo Sawatani, Masahiko Murakami

**Affiliations:** Esophageal Cancer Center, Showa University Hospital, Tokyo, Japan

**Keywords:** Bypass surgery, Esophageal cancer, Thoracoscopic drainage

## Abstract

**Background:**

Esophagogastric bypass is performed for esophageal strictures. Mucus retention, known as mucocele, sometimes occurs at the stricture oral side of the remnant esophagus. It is often asymptomatic and is expected to be naturally decompressed, but it may cause respiratory failure depending on the case. Herein, we report a case in which we successfully performed thoracoscopic esophageal drainage as emergency airway management due to tracheal compression by a mucocele after esophagogastric bypass for unresectable esophageal cancer with esophagobronchial fistula.

**Case presentation:**

A 56-year-old man underwent esophageal bypass surgery for an unresectable esophageal carcinoma with an esophagobronchial fistula following chemotherapy and radiation therapy. Nine months after bypass surgery, he experienced severe dyspnea due to tracheal compression caused by mucus retention on the oral side of the esophageal tumor. We planned thoracoscopic surgery for mucus retention drainage through the right thoracic cavity to secure the airway as an emergency procedure under general anesthesia. Intubation can be performed safely by guiding bronchoscopy in the semi-supine position. Upper esophageal dilation was observed on the cranial side of the azygos arch. We dissected the mediastinal pleura of the upper thoracic esophagus and exposed its wall. A 12-Fr silicone drain was placed in the esophagus through the right chest wall and 120 ml of white fluid was aspirated. He was discharged 9 days after surgery without complications and resumed treatment with an immune checkpoint inhibitor 23 days after surgery. Thereafter, he continued chemotherapy for esophageal cancer, but died of tumor progression and lung metastasis 35 months after bypass surgery and 25 months after thoracoscopic surgery.

**Conclusions:**

Thoracoscopic esophageal drainage could be performed safely as emergency airway management, shorten the period of discontinuance, and allow cancer treatment to be resumed promptly. We believe that this thoracoscopic procedure is an effective and less invasive method if the percutaneous approach is difficult.

## Background

Esophagogastric bypass is performed for esophageal strictures due to malignancy. During esophageal bypass surgery, the gastric tube pulled up through the retrosternal route to the neck is commonly anastomosed to the cervical esophagus through a left neck incision. Esophageal bypass surgery for malignant esophageal stricture is performed to improve quality of life by allowing oral intake for unresectable advanced esophageal cancer [1, 2]. It is sometimes necessary to perform drainage to prevent mucus retention in the remnant esophagus (known as mucocele), because it is usually asymptomatic but sometimes causes respiratory failure [3, 4]. Herein, we report a case in which we performed thoracoscopic esophageal drainage as emergency airway management caused by tracheal compression due to a mucocele on the oral side of the esophageal tumor after esophagogastric bypass with external drainage for unresectable esophageal cancer.

## Case presentation

**Fig. 1:**
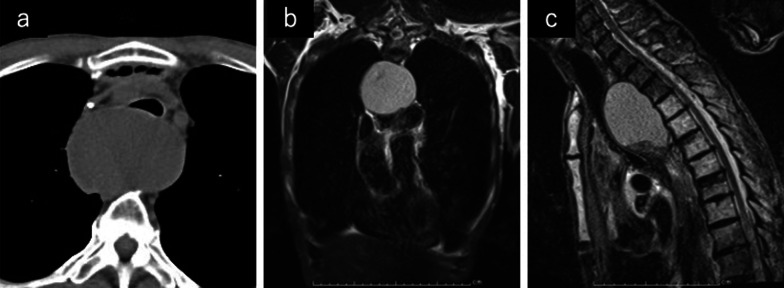
Mucocele of the esophagus. **a** CT scan showing tracheal compression due to the mucocele. **b** MRI showing the presence of fluid collection and dilatation of the remnant esophagus. **c** MRI showing tracheal compression due to the mucocele on the sagittal view

**Fig. 2:**
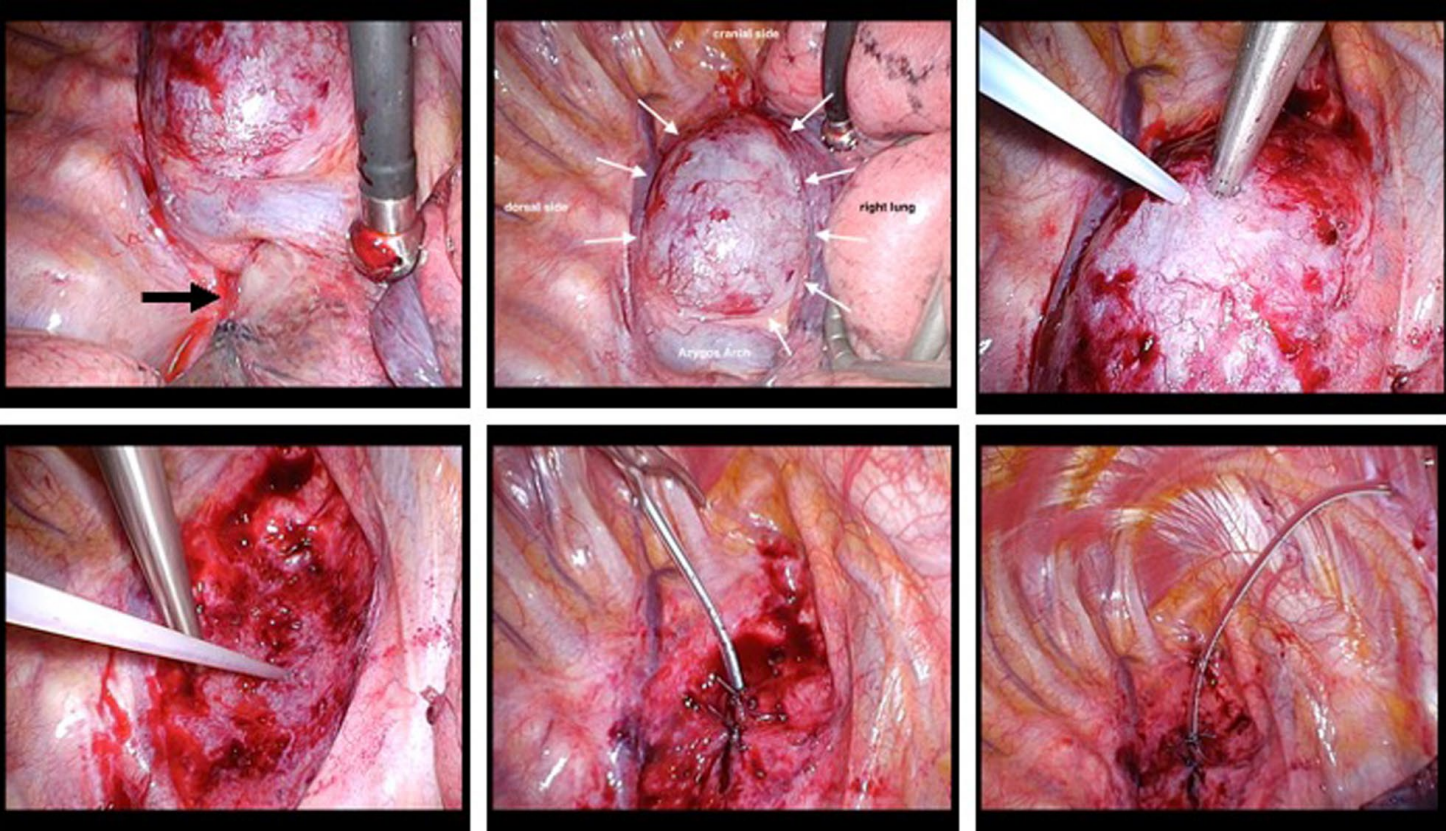
Surgical findings. Upper esophageal dilation was shown on the cranial side of the azygos arch (white arrow), and a tumor was found at the level of the tracheal bifurcation (black arrow). A fine needle puncture was performed, and a white-colored fluid was aspirated. A 12-Fr silicone drain was inserted from the right chest wall. The drain was fixed at the esophageal wall and closed the mediastinal pleura covering it

A 56-year-old man presented at our hospital with dysphagia. He was diagnosed with unresectable advanced esophageal squamous cell carcinoma with mediastinal lymph node metastasis and esophagobronchial fistula. The tumor formed a very strong stenosis, which did not allow a narrow-bore endoscope to pass through. He underwent esophagogastric bypass surgery comprising anastomosis between the cervical esophagus and subtotal gastric tube, pulling up through the retrosternal route with external drainage of the remnant esophagus using a silicone tube from the abdominal wall. Subsequently, he was able to have meals and underwent chemotherapy and therapy with immune checkpoint inhibitors following chemoradiotherapy. Nine months after surgery, he was admitted to our hospital with severe dyspnea and hoarseness and could not be kept in supine position. Computed tomography (CT) and magnetic resonance imaging (MRI) revealed marked dilation of the residual esophagus on the oral side of the tumor and heavy tracheal exclusion (Fig. 1). Thoracoscopic surgery was planned to secure the airway during the emergency surgery. Intubation can be performed safely by guiding bronchoscopy in the semi-supine position. We prepared extracorporeal membrane oxygenation for use when needed. He was placed in the left lateral decubitus position, and thoracoscopic surgery was performed with five ports and one-lung ventilation. There were adhesions between the mediastinal pleura and the right lung in the upper mediastinum, which were dissected safely. Upper esophageal dilation was observed on the cranial side of the azygos arch, and a tumor was found at the level of the tracheal bifurcation. We dissected the mediastinal pleura of the upper thoracic esophagus and exposed its wall. Subsequently, a puncture using a fine needle was performed, and 120 ml of white fluid was aspirated. A 12-Fr silicone drain was inserted from the right chest wall below the right subclavian artery along the chest wall and was placed in the esophagus. The drain was fixed to the esophageal wall, and the mediastinal pleura was closed (Fig. 2). The drainage tube was fixed to the esophageal wall using a non-absorbable suture; subsequently, we fixed mediastinal pleura and esophageal wall covering the drainage tube similar to that in Witzel’s method. This surgery lasted 90 min.

**Fig. 3:**
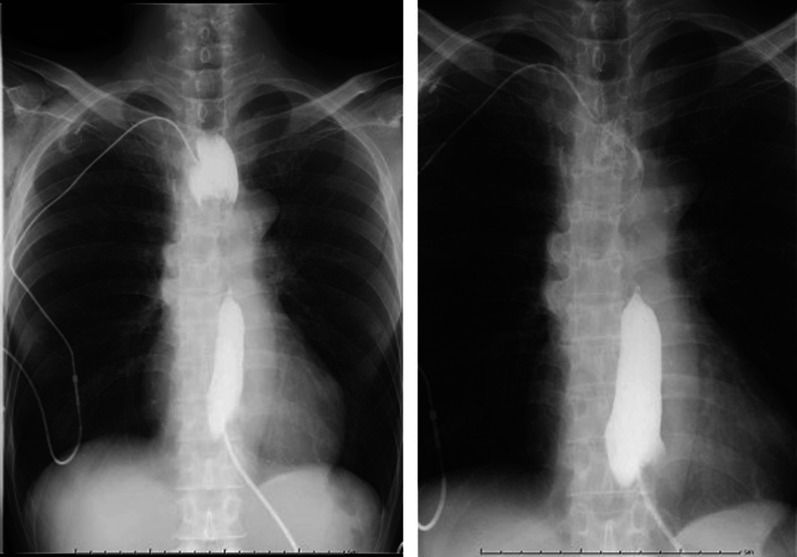
X ray findings after esophageal drainage. A drain contrast study was performed and showed that dilatation of the upper thoracic esophagus had disappeared

**Fig. 4:**
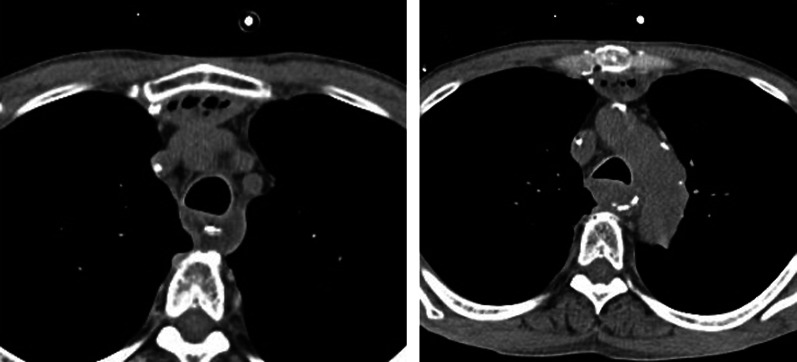
Findings of CT after esophageal drainage. The CT scan showed that the mucocele was still absent 2 months after the surgery

The stenosis of the trachea and hoarseness disappeared, and the patient was able to maintain a supine position without dyspnea after surgery. A day after the surgery, a drain contrast study was performed, which showed the dilatation disappeared (Fig. 3). The patient was discharged 9 days after surgery without complications. He resumed treatment with an immune checkpoint inhibitor 23 days after the surgery. A CT scan showed that the mucocele had disappeared 2 months after surgery (Fig. 4). Three months later, a bronchial stent was inserted for invasion of the left bronchus due to tumor progression. Radiotherapy for brain metastasis was performed 13 months later. The patient continued chemotherapy for esophageal cancer. However, the patient died of tumor progression and lung metastasis 35 months after bypass surgery and 25 months after thoracoscopic surgery.

## Discussion

Esophageal mucocele is a rare complication of esophageal diversion procedures that lead to the formation of a blind esophageal loop, and persistent secretions from the mucosa lead to the development of mucocele [5]. This can be diagnosed by its high‑intensity signal in T2‑weighted MRI [3, 5]. Based on previous reports, mucocele was detected after performing esophageal bypass in patients who underwent surgery for benign conditions, such as perforation, corrosive stricture, achalasia, and carcinoma [3–10]. Collins et al. showed percutaneous drainage with absolute alcohol ablation for mucocele [11]. A previous study reported a mucocele after esophageal bypass surgery, which was drained through R–Y loop of jejunum [6]. However, the treatment of mucocele was commonly performed with a thoracotomy including remnant esophagectomy or mucosectomy [3–5, 7, 8, 10]. In recent years, the thoracoscopic surgery was being performed in this condition [9], but we found no reports of endoscopic treatment for mucocele after esophageal bypass. Mucocele is often asymptomatic and is expected to be naturally decompressed [6, 10]. However, it should be noted that there are reports of severe respiratory disorders due to mucocele [3, 4].

Esophageal bypass for advanced esophageal cancer with severe stenosis is one of the procedures that facilitates oral food intake [1, 2]. Mucocele sometimes occurs at the oral side of the stricture of the remnant esophagus [1]. It is important to prevent mucocele at the oral side of the esophageal stricture, and the fluid should be drained. It is also necessary and important to avoid rupture of the esophageal stump caused by mucocele. Internal drainage is known as the Postlethwait method [12] and Kirschner method [13], and external drainage is performed using a tube inserted from the abdomen [14]. Cardiostomy is also known as one of the external drainages [15]. As patients sometimes need artificial ventilation support if an esophagobronchial fistula grows, external drainage is sometimes better than internal drainage in these patients [2, 14]. Therefore, the surgical procedure should be selected based on the case.

In this case, airway compression by mucocele that occurred in the residual esophagus caused severe dyspnea. A percutaneous approach was not used because of anatomical difficulty. The cervical approach was not adopted because of the position of the brachiocephalic artery. The intercostal approach from lateral vertebral body was also considered, but there was a risk for injury of right lung and the drainage tube needs to be inserted in this approach. Thus, we considered that the precordial approach is better for drain management than other approaches. Although a tracheal stent was considered, it was not used due to risk for rupture, because hoarseness had not improved. Therefore, thoracoscopic esophageal drainage was performed. We were concerned that esophageal leakage would cause bacterial infection in the thoracic cavity and carcinomatous pleurisy following drainage. However, the tumor was controlled with multimodal therapy, and selecting a reliable and prompt improvement method was necessary. In this case, external drainage could not be performed from the distal side of the stricture during the bypass surgery. Therefore, it may be desirable to create a cervical esophageal fistula.

A disadvantage of this surgical procedure is that the drainage tube remains on the body surface. An external esophageal drainage was already present in the abdominal wall during the bypass surgery, and an additional drainage was added to the precordium. The patient often had to suck the fluid as appropriate. While the patient sometimes sucks the fluid in the mucocele of the esophagus, the drainage did not require replacement until the drainage tube was lost at 12 months after this surgery. However, the mucocele did not require drainage subsequently. In this case, we chose this procedure as an emergency surgery and was successfully performed.

Esophageal cancer with esophagobronchial fistula has a poor prognosis [2]. This patient underwent multimodal treatment, including chemotherapy, radiotherapy, and therapy with immune checkpoint inhibitors after esophageal bypass surgery for locally advanced esophageal cancer and metastasis. The patient survived for more than 30 months after bypass surgery. He had to temporarily abandon cancer treatment by esophageal mucocele, but thoracoscopic drainage could shorten the period of discontinuance and allow the cancer treatment to be resumed promptly.

## Conclusions

We experienced a case in which we performed thoracoscopic esophageal drainage for a mucocele on the oral side of the esophageal tumor after esophagogastric bypass for unresectable esophageal cancer. Thoracoscopic esophageal drainage could be performed safely as emergency airway management, shorten period of discontinuance, and allow cancer treatment to be resumed promptly. We believe that this thoracoscopic procedure is an effective and less invasive method if the percutaneous approach is difficult.

## Data Availability

Date sharing is not applicable to this article.

## Abbreviations

CT: Computed tomography
MRI: Magnetic resonance imaging

## References

[1] Conlan AA, Nicolaou N, Hammond CA, Pool R, de Nobrega C, Mistry BD (1983). Retrosternal gastric bypass for inoperable esophageal cancer: a report of 71 patients. Ann Thorac Surg.

[2] Nakajima Y, Kawada K, Tokairin Y, Miyawaki Y, Okada T, Miyake S, Kawano T (2016). Retrospective analyses of esophageal bypass surgery for patients with esophagorespiratory fistulas caused by esophageal carcinomas. World J Surg.

[3] Haddad R, Teixeira Lima R, Henrique Boasquevisque C, Antonio MG (2008). Symptomatic mucocele after esophageal exclusion. Interact Cardiovasc Thorac Surg.

[4] Lee SY (2005). Tracheal compression by esophageal mucocele after surgical exclusion of the esophagus. Eur J Cardiothorac Surg.

[5] Tewari S, Goyal P, Rastogi A, Agarwal A, Singh PK (2017). Anesthetic challenges of extrinsic trachea-bronchial compression due to posterior mediastinal mass: our experience with a large esophageal mucocele. Ann Card Anaesth.

[6] Olsen CO, Hopkins RA, Postlethwait RW (1985). Management of an infected mucocele occurring in a bypassed excluded esophageal segment. Ann Thorac Surg.

[7] van Till JW, van Sandick JW, Cardozo ML, Obertop H (2002). Symptomatic mucocele of a surgically excluded esophagus. Dis Esophagus.

[8] Manickam Neethirajan S, Chandramohan SM, Velayoudam V, Aridhasan Meenakshi L, Harikrishnan S (2019). Giant mucocele of the remnant esophagus: case report of a rare complication following a bipolar esophageal exclusion procedure. Cureus.

[9] Sapkota R, Thapa B, Sayami P (2019). Esophageal ‘pyocele’: thoracoscopic management. J Surg Case Rep.

[10] Kamath MV, Ellison RG, Rubin JW, Moore HV, Pai GP (1987). Esophageal mucocele: a complication of blind loop esophagus. Ann Thorac Surg.

[11] Collins KC, Odell DD, Sheiman RG, Gangadharan SP (2012). Critically compromised airway secondary to expanding esophageal mucocele. Ann Thorac Surg.

[12] Postlethwait RW (1979). Technique for isoperistaltic gastric tube for esophageal bypass. Ann Surg.

[13] Ong GB (1973). The Kirschner operation—a forgotten procedure. Br J Surg.

[14] Ohsawa M, Hamai Y, Ibuki Y, Emi M, Miyata Y, Okada M (2019). Long-term esophageal cancer survivor treated by bypass for esophagobronchial fistula after chemoradiotherapy: a case report. Anticancer Res.

[15] Seto Y, Yamada K, Fukuda T, Hosoi N, Takebayashi R, Chin K, Kotsuka T, Gomi K, Yamaguchi T (2007). Esophageal bypass using a gastric tube and a cardiostomy for malignant esophagorespiratory fistula. Am J Surg.

